# Efficient Solar-Driven Water Purification Based on Biochar with Multi-Level Pore Bundle Structure for Preparation of Drinking Water

**DOI:** 10.3390/foods10123087

**Published:** 2021-12-13

**Authors:** Zhen Zhang, Shizheng Jiang, Haonan Chen, Hao Qi, Yali Chen, Yujie Chen, Qiliang Deng, Shuo Wang

**Affiliations:** 1 State Key Laboratory of Food Nutrition and Safety, Key Laboratory of Marine Resource Chemistry and Food Technology (TUST), Ministry of Education, Tianjin University of Science and Technology, Tianjin 300457, China; zhangzhen163zz@163.com (Z.Z.); jsz256112@163.com (S.J.); chenhaonan_1997@126.com (H.C.); kylin57796@163.com (H.Q.); 18622666229@163.com (Y.C.); 18222936434@163.com (Y.C.); yhdql@tust.edu.cn (Q.D.); 2 Tianjin Key Laboratory of Food Science and Health, School of Medicine, Nankai University, Tianjin 300071, China

**Keywords:** drinking water, sorghum stalk, carbonized, high efficiency, solar steam generation

## Abstract

Water is an important source for humankind. However, the amount of available clean water has rapidly reduced worldwide. To combat this issue, the solar-energy-driven evaporation technique is newly proposed to produce clean water. Here, biochar derived from sorghum stalk with a multi-level pore bundle structure is utilized to fabricate a solar-driven evaporator for the first time. The biochar displays rapid water transfer and low thermal conductivity (ca. 0.0405 W m^−1^ K^−1^), which is vitally important for such an application purpose. The evaporation rate and energy conversion efficiency of the solar evaporator based on carbonized sorghum stalk can achieve up to 3.173 kg m^−2^ h^−1^ and 100%, respectively, which are better than most of the previously reported biomass materials. Furthermore, the carbonized sorghum stalk also displays good resistance to salt crystallization, anti-acidic/basic, and organic pollutants by producing drinking water using seawater, acidic/basic waste water, and organic polluted water, respectively. The direct application of processed water in food production was also investigated. The present solar steam evaporator based on the carbonized sorghum stalk has the potential to create practical drinking water production by using various water sources.

## 1. Introduction

As the source of life, water is the basic material for human survival. In addition, water is also an important part of food and raw material in the production and processing of other food. However, water contamination and drinking-water scarcity have become ever-present, increasing the global challenges facing human society as modern industry and urbanization rapidly develop due to population growth [1,2]. Two out of three people will suffer from the shortage of freshwater by 2025 [3]. The desalination and purification of seawater and wastewater are considered as key ways to overcome the issue [4,5]. A variety of advanced technological means have been exploited to produce drinking water from seawater and wastewater. For example, multi-stage flash desalination (MSF) is a mature phase change method which accounts for 64% of the thermal desalination or distillation technologies. Although MSF is reliable for large-scale seawater desalination, the equipment is expensive and the process is energy intensive [6]. Microbial desalination cells (MDCs) are promising technologies for addressing the shortage of water resources, which show energy savings compared with MSF. However, the issues of membrane biofouling, pH value fluctuation, and the internal resistance limit its further large-scale usage [7]. Multi-effect distillation (MED) is the first commercialized thermal desalination technology. MED technology is mature, and has scaling issues and high operating costs [8]. Pressure-retarded osmosis (PRO) can harvest the Gibbs free energy produced by the mixing of different salinities streams and further convert it to electric energy. Although the PRO technology has been improved in the past few years, the issues of membrane material and pollution, efficiency, water conveyance in the membrane and techno-economic feasibility are still unresolved [9]. Nuclear desalination is an emerging desalination technology; nuclear energy can be used as an alternative to fossil fuels to drive thermal desalination and the cost is acceptable. However, some safety concerns impede the development of nuclear desalination [8]. In addition, other technologies such as membrane technology [10], reverse osmosis technology [11], ultrafiltration [12], advanced oxidation [13], electrodialysis [14], and capacitive deionization (CDI) [15] have also demonstrated the limited application potential due to the high cost and environmentally unfriendly process [16,17]. Therefore, effective, simple-to-operate, low-cost, and eco-friendly technology is urgently needed to provide clean water.

Solar energy is a green, inexhaustible, and renewable energy source with no region restrictions [18,19]. The production of drinking water by solar-driven steam generation is attracting increasing attention from researchers and business people [20,21]. The efficiency of solar steam generation mainly depends on high-performance photothermal materials. Synthetic materials such as plasmonic materials [22], molybdenum nitride [23], mixed metal oxide [24], porous polymer foam [25] and carbon nanotubes [26] have been devoted to designing and fabricating the device for such an application. To some extent, these materials were successful; however, the questionable environmental friendliness of the preparation procedure and the high-cost raw materials significantly hinder their large-scale application. Most biomass materials possess complex microstructures and multi-level structures, which facilitate both water absorption and transport [17,27]. Taking full advantage of their unique native structures, low cost, huge availability, rapid regeneration, and eco-friendly features, biomass materials such as carbonized corncob [28], surface-carbonized saccharum [29], and wood [30] have been investigated as solar steam generators. Compared with the synthetic materials, the performance of the previously reported biomass materials is still unsatisfactory due to their lower thermal management or/and water absorption/transport ability [31,32]. Therefore, the exploitation of a biomass material that simultaneously combines good thermal control with high-efficiency water transportability is vitally important.

Sorghum, as one of the five most important cereal crops, has a short growth period (3–5 months) [33,34] and is widely cultivated in tropical, subtropical and temperate regions [35]. As a vascular plant, there are abundant vascular bundles in the sorghum stalk. These vascular bundles play important roles in the transportation of nutrients and facilitate the rapid transportation of water. This feature makes it suitable for the use as a solar evaporator. Here, a solar steam generator based on sorghum stalk is constructed for the first time. Using a simple carbonizing treatment, the carbonized sorghum stalk shows a black surface, and the pore structure is similar to the original one. The structure and composition of the carbonized sorghum stalk were characterized. The evaporation performance was investigated under solar light irradiation with an intensity of 1 kW m^−2^. The processed seawater was also directly applied for food production. The present evaporator, based on the carbonized sorghum stalk, opens a novel avenue for the prospective optimization and fabrication of high-efficiency solar steam evaporators.

## 2. Materials and Methods

### 2.1. Materials Preparation

Sorghum straw was obtained from cropland after the harvest (Jinan, China), which was then cut into different heights. The preparation process of carbonized sorghum straw was based on the previous literature with some improvements [3,29,36]. In brief, the sorghum straw was firstly pre-frozen at −20 °C for 12 h, and then treated by freeze-drying at −80 °C for 48 h. Finally, the dried straw was heated with a tube furnace in Ar condition from 20 °C up to 400 °C. The heating rate and processing time was 5 °C min^−1^ and 2 h, respectively.

### 2.2. Characterization

The scanning electron microscope (SEM) was used to characterize the microscopic morphologies of the carbonized sorghum straw (SM-IT300LV, JEOL, Tokyo, Japan). The mercury porosimeter was used to characterize the pore size distribution of the carbonized sorghum straw (AutoPore IV 9510, Micromeritics, Atlanta, GA, USA). A Fourier transform infrared spectrometer (FT-IR) was used to characterize the functional groups (TENSOR27, Bruker, Karlsruhe, Germany). X-ray photoelectron spectroscopy (XPS) was applied to characterize the surface compositions of the carbonized sorghum straw (250Xi, Thermo ESCALAB, Waltham, MA, USA) An ultraviolet-visible–near-infrared spectrometer (UV-vis-NIR) was used to measure the optical reflectance (R) and transmittance (T) spectra of the carbonized sorghum straw (UV 3600 Plus, Shimadzu, Kyoto, Japan), and the sample absorption was calculated from A = 1 − R − T. The transmittance of the carbonized sorghum straw was set to zero. X-ray diffractometer was applied to measure X-ray diffraction (XRD) patterns of the carbonized sorghum straw (D8 ADVANCE, Bruker, Karlsruhe, Germany). The Raman spectra of the carbonized sorghum straw were determined by a Raman microscope (HR800, HORIBA JY, Kyoto, Japan). The water contact angles of the carbonized sorghum straw were characterized by a video optical contact angle measuring device (DSA30, KRUSS, Hamburg, Germany). The thermal conductivities of natural and carbonized sorghum straw were measured by a thermal conductivity measuring apparatus (TC3000E, XIATECH, Xian, China).

### 2.3. Solar Evaporation Performance Tests

The solar evaporation performance tests were performed on a solar simulator (PLS-SXE300D, Perfect Light, Beijing, China). The power meter (PM100D, THORLABS, Bremen, Germany) was used for measuring solar flux. The mass loss of water was monitored by an electronic balance (Quintix124, Sartorius, Gottingen, Germany). An infrared (IR) camera was used to record the temperature changes in the samples (226s, FOTRIC, Shanghai, China). The energy conversion efficiency (η) is given by Equation (1) [29]:

(1) $$\eta =\frac{{\mathrm{mh}}_{\mathrm{lv}}}{I}$$

m: evaporation rate;
I: power density of simulated solar illumination;
h_lv_: total enthalpy including phase change (h_lv(T)_) from liquid water to vapor and sensible heat (Q) can be calculated by the following equations [18]:

h_lv(T)_ can be calculated by Equation (2):

(2) $$h_{\mathrm{lv}\left(T\right)}=1.91846\times 10^{6}{\left[T/\left(T-33.91\right)\right]}^{2}$$

T: average surface temperature of the evaporator.
Q can be calculated by Equation (3):
  (3) $$Q=c\left(T-T_{1}\right)$$
C: specific heat of water;
T_1_: initial temperature of water.

The evaporation rate in darkness was subtracted.

## 3. Results and Discussion

### 3.1. Microstructure and Chemical Composition Characterization

**Scheme 1:**
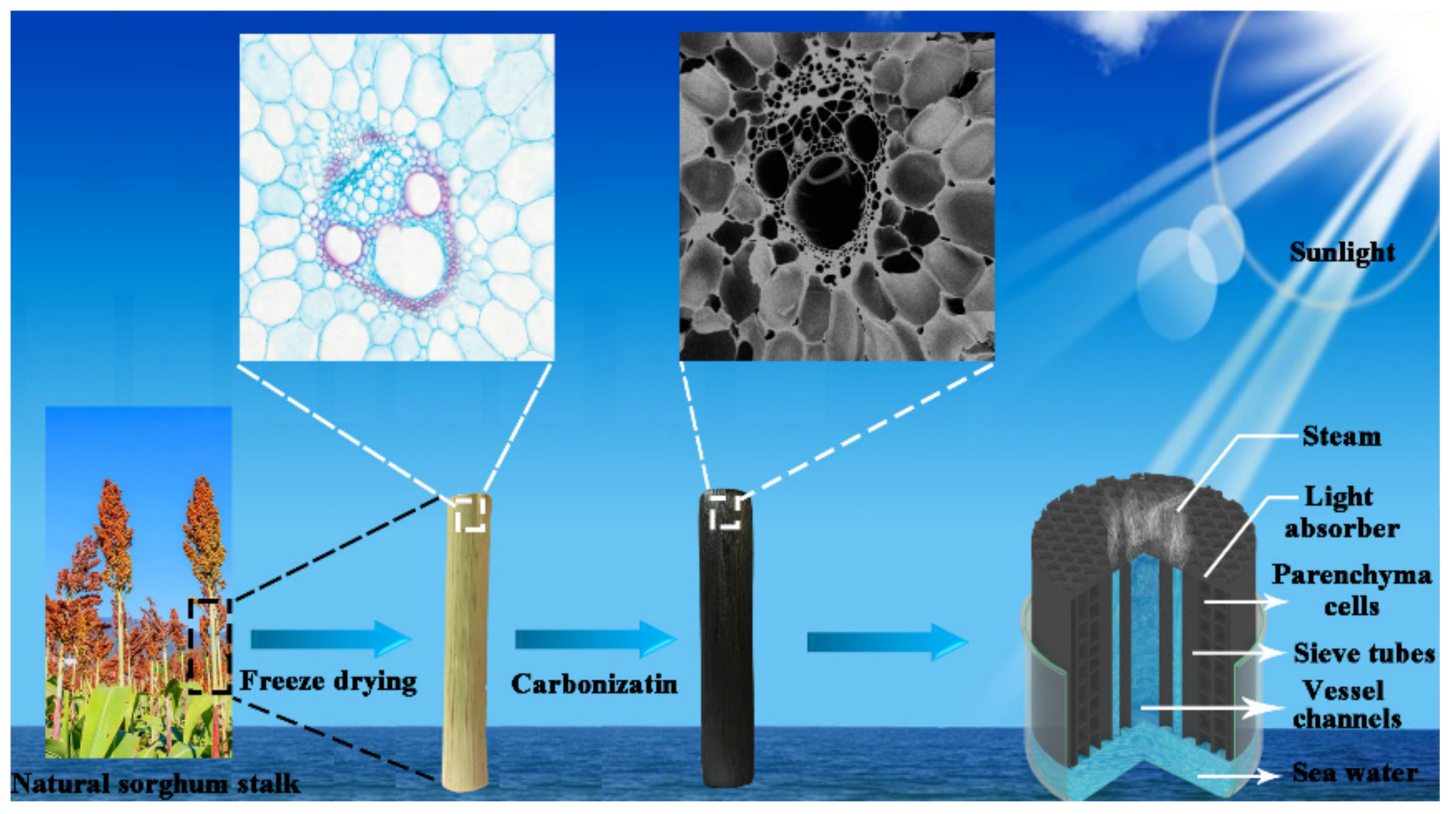
Construction and schematic process of the carbonized sorghum straw for solar evaporation.

Carbonized sorghum straws were obtained by the following three main steps. Firstly, the different heights of the sorghum straws (1, 2, 3, 4, 5, 6, 7, 8, 9, and 10 cm) were obtained by cutting along their growth directions. The preparation process is shown in Scheme 1.

**Figure 1:**
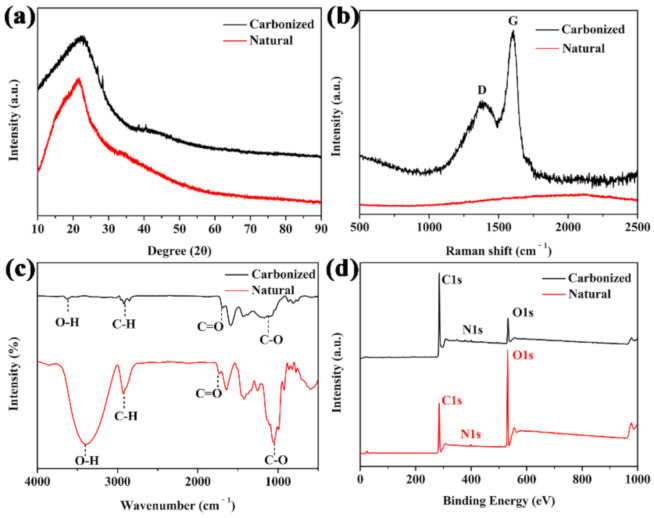
(**a**) XRD pattern, (**b**) Raman spectrum, (**c**) FT-IR spectra, and (**d**) XPS survey spectra of natural and carbonized sorghum straws.

In order to characterize the properties of the carbonized materials, the XRD analysis was performed. The carbonized sorghum straw showed a wider peak than that of the natural one, and the peak positions had no significant shift. The results indicated that the carbonized material was mainly composed of amorphous carbon [37] (Figure 1a). The Raman spectrum presented that the broad disorder-induced band (D band) at ~1360 cm^−1^ and the in-plane vibrational band (G band) at ~1590 cm^−1^ (Figure 1b) revealed amorphous carbon and sp2 vibration of graphite crystal [38,39]. The functional groups of natural and carbonized sorghum straw were firstly analyzed by FT-IR. Most of the hydrophilic groups in the natural sorghum straw were retained in the carbonized sorghum straw (Figure 1c).

The peaks of O-H bonds in natural and carbonized sorghum straw were observed at 3409 and 3615 cm^−1^, respectively. The peaks at around 2925 and 2919 cm^−1^ were attributed to C-H bonds. The peaks at 1737 and 1692 cm^−1^ derived from a C=O stretching vibration. The representative peaks of the C-O bonds were observed at 1051 and 1120 cm^−1^. The existences of these hydrophilic groups demonstrated the hydrophilicity of the carbonized sorghum straw [40]. The surface compositions of natural and carbonized sorghum straw were then analyzed by XPS. As shown in Figure 1d, C 1s and O 1s peaks were found in both samples at 285.7 and 531.9 eV, respectively. A weak N 1 s peak was observed at 400.6 eV. These residual nitrogen species endowed the carbonized sorghum straw with the hydrophilicity [41]. The elemental content of O decreased from 37.9 to 12.3%. On the contrary, the elemental content of C increased from 60.4 to 85.8%, revealing that deoxidation reactions occurred [3].

**Figure 2:**
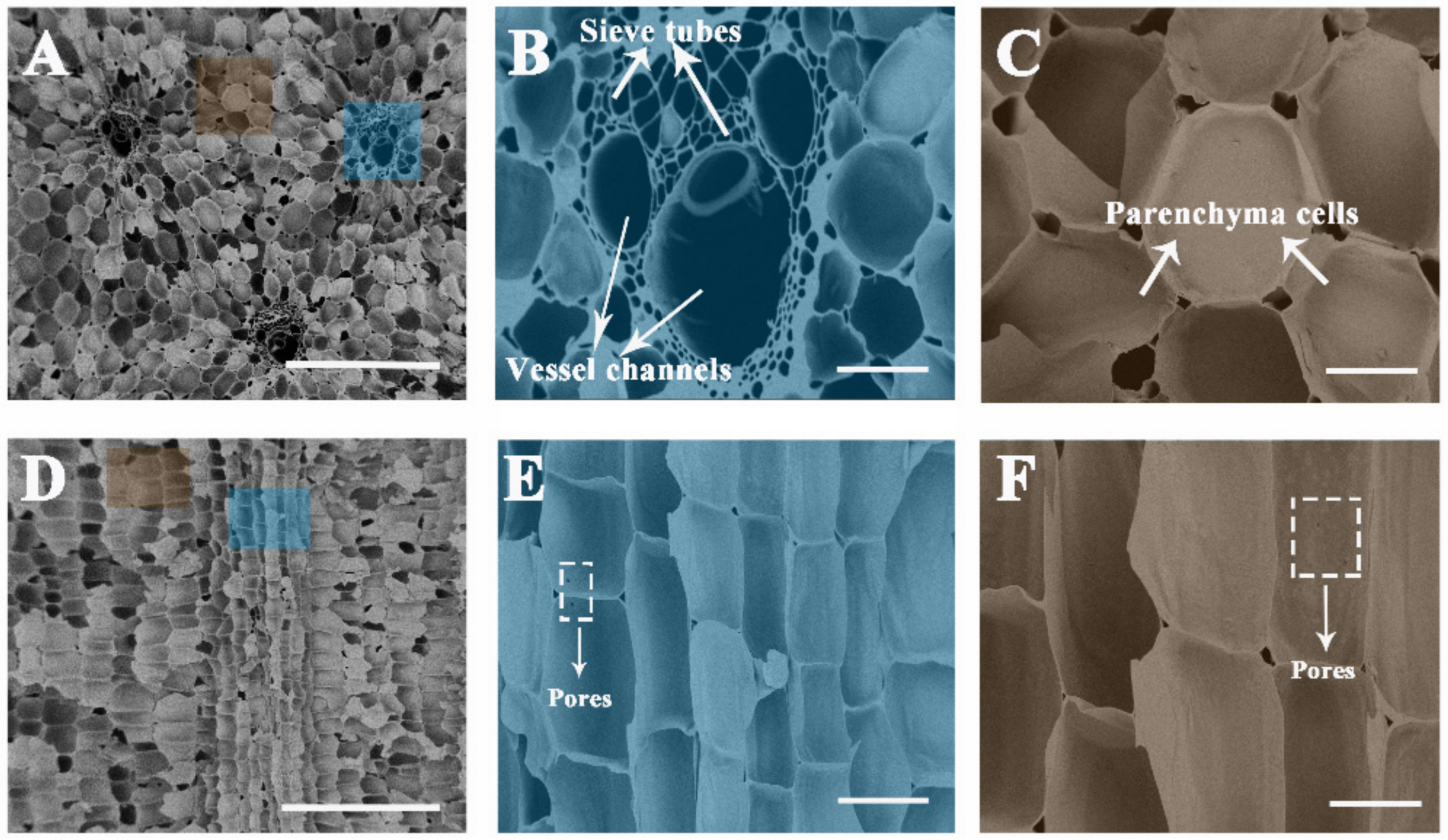
SEM images of the carbonized sorghum straw. (**A**–**C**) Cross section and (**D**–**F**) vertical section of the sample. Scale bars: 500 μm (**A**,**D**); 100 μm (**B**,**C**); 50 μm (**E**,**F**).

The optical microscope images of the natural sorghum straw showed a uniform distribution of multi-level pore bundles (Figure S1a). Figure S1b is the typical image of a vascular bundle, which consists of vessel channels and sieve tubes. Parenchyma cells line up neatly around the vascular bundles (Figure S1c). After carbonization, the above structures were retained. The SEM images revealed that the vascular bundles were constructed by vessel channels (ca. 50–100 μm) and sieve tubes (ca. 2–20 μm) (Figure 2A). Numerous thin sieve tubes were arranged around these vessel channels (Figure 2B). The vessel channels formed centralized water conveyance units and acted as freeways to highly efficient water transport for evaporation. Additionally, the parenchyma cells (ca. 10–100 μm in diameter) arranged continuously between the multi-level pore bundles (Figure 2C), which can conduce to higher solar absorption by reducing light reflection and enhancing light scattering effects [29,37]. Interestingly, there were ca. 5–10 μm-sized pores distributed between the neighbor parenchyma cells (Figure 2C). The pore size distribution of the carbonized sorghum straw was further investigated by a mercury porosimeter. Macropores (ca. 3–130 μm in diameter) were measured (Figure S2), which accord with the sieve tubes, vessel channels, and pores between the neighbor parenchyma cells, respectively.

The longitudinal section morphologies of natural and carbonized sorghum straws are shown in Figure S1d and Figure 2D, respectively. The narrow structure of sieve tubes provided strong capillarity rising and sufficient water supply. From the longitudinal section view, the sieve tubes and parenchyma cells were connected by pores (ca. 2 μm in diameter) (Figure 2E,F).

**Figure 3:**
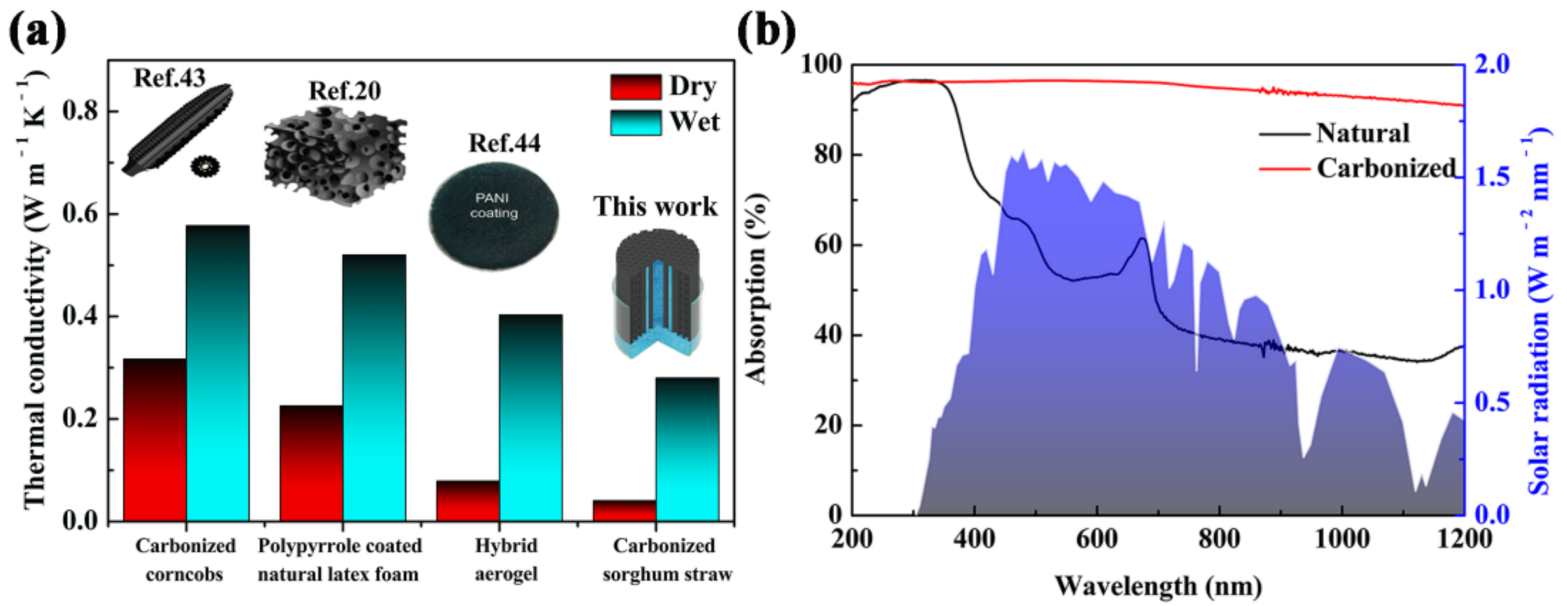
(**a**) Comparison of thermal conductivity with reported materials. (**b**) Absorption spectra of natural and carbonized sorghum straws and solar spectral irradiance (AM 1.5G, shown by the blue area).

The part composed of parenchyma cells was nearly an enclosure space filling with air, which can isolate water from the ambient environment and ensure its low thermal conductivity (ca. 0.0405 W m^−1^ K^−1^). In terms of thermal conductivity, the carbonized sorghum straw was similar to polystyrene foam (0.04 W m^−1^ K^−1^), and was significantly superior to water (0.60 W m^−1^ K^−1^) [41,42]. Consequently, the carbonized sorghum straw as a prominent thermal barrier was suitable for a solar evaporator. Furthermore, the thermal conductivity of the wet carbonized sorghum straw was improved to ca. 0.28 W m^−1^ K^−1^. This result was due to the hydrophilicity of the carbonized sorghum straw. Nevertheless, the enclosure space filling with air in carbonized sorghum straw endowed satisfied a heat-insulating property of the wet carbonized sorghum straw. Compared to previously reported literature, the carbonized sorghum straw exhibited the lowest thermal conductivity (Figure 3a), which was helpful to reduce the thermosteresis from evaporation interfaces to bulk water [20,43,44].

Light absorption of the carbonized sorghum straw is one of the key factors for solar evaporation. The light absorption of natural and carbonized sorghum straws was measured by UV-vis-NIR spectroscopy. Compared with the natural sorghum straw, the carbonized sorghum straw showed a higher absorptance, ranging from 200 to 1200 nm (Figure 3b). Weighted with air mass 1.5 global (AM 1.5G) solar spectrum, the carbonized sorghum straw showed a high light absorptance (96.4%) within the full wavelength range. For the natural sorghum straw, the average light absorptances were only ca. 56.3% in the visible region and ca. 36.6% in the near-infrared region. The remaining inorganic substance may have resulted in less than 100% absorbance of the carbonized sorghum straw [38]. The light reflectance of carbonized sorghum straw was lower than that of the natural carbonized sorghum straw in the whole wavelength range, which resulted in the above results (Figure S3).

The hydrophilicity of carbonized sorghum straw was a significant characteristic, allowing water to transport from the bottom to the evaporation interface. The contact angle of the carbonized sorghum straw was nearly 0°, which was attributed from an abundant vascular bundle structure and hydrophilic groups (Figure S4). Consequently, the pores on the intine of the sieve tubes and parenchyma cells exhibited the lateral wetting property, contributing to the abundant water supply. To further estimate the hydrophilicity of the carbonized sorghum straw, a carbonized sorghum straw was placed in water and its top was covered with a piece of filter paper. As shown in Figure S5, the water was quickly transported from the bottom of the carbonized sorghum straw to the evaporation interface within 10 s and the filter paper was wetted within 60 s. The rapid water transport effectively promoted the solar evaporation.

### 3.2. Optimization of the Carbonized Sorghum Straw’s Height

**Figure 4:**
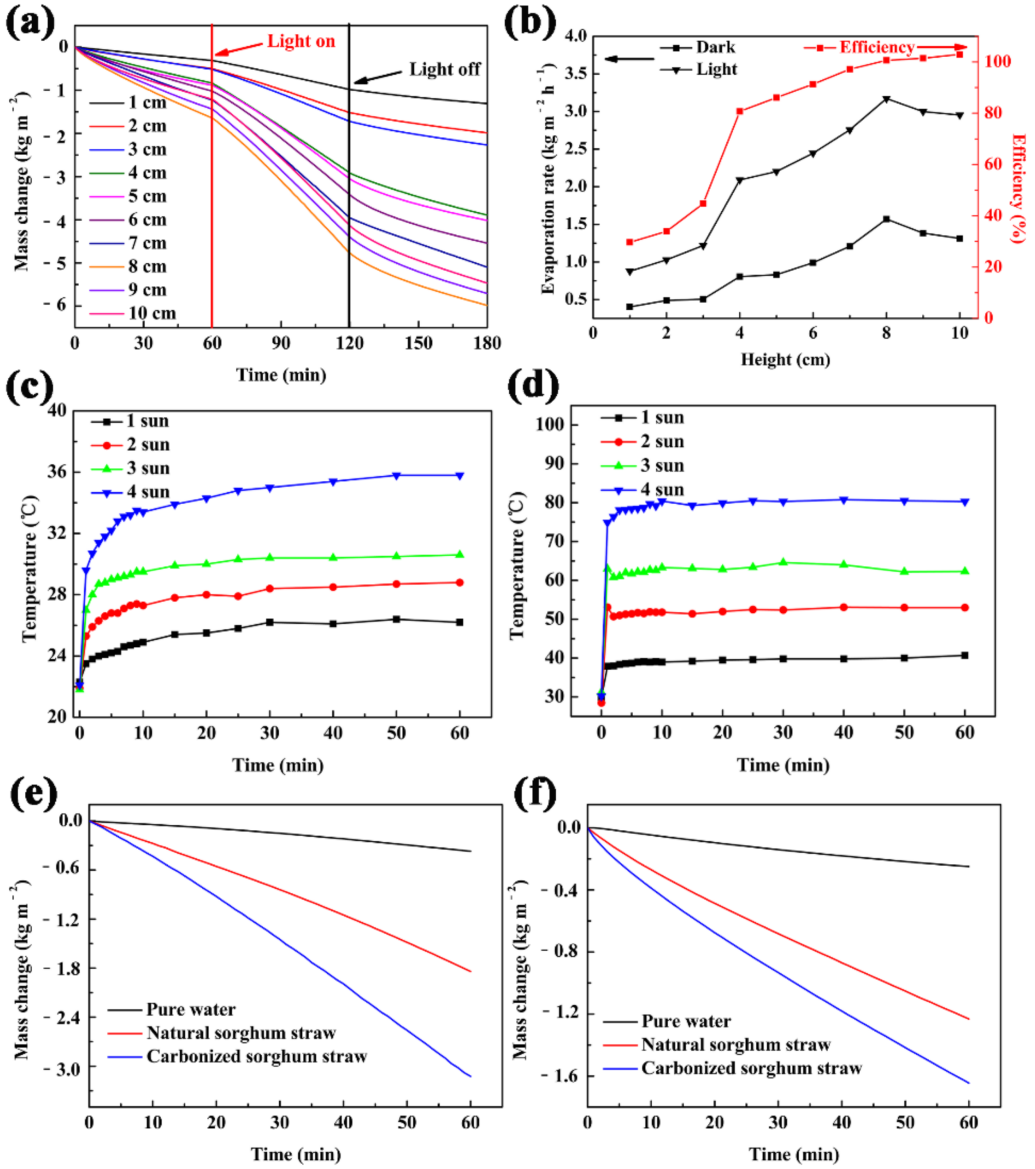
(**a**) Mass change, (**b**) Evaporation rates and efficiency of the carbonized sorghum straws with different height. Temperature changing curves of wet (**c**) and dry (**d**) carbonized sorghum straws. Mass change of pure water, natural, or carbonized sorghum straw under 1 Sun illumination (**e**) or in the dark (**f**). (**a**–**e**) were operated under 1 Sun illumination.

To optimize the water transport distance during purification, the evaporation rates and efficiency of the carbonized sorghum straws with different heights were systematically studied under 1 Sun (1 kW m^−2^) illumination for 1 h. The mass changes sped up with the increase of the carbonized sorghum straw height, in the range of 1 cm to 8 cm, which can be attributed to the side wall gaining energy from the environment to drive water evaporation (Figure 4a) [45,46,47]. The evaporation rate did not continue to speed up as the carbonized sorghum straw height increased. It was attributed that the higher sorghum straw led to the increased distance that water was transferred, and the overlong water transfer distance decreased the water supply and suppressed the water evaporation [29].

The results of the evaporation rates and efficiency well-matched the mass change results (Figure 4b). In addition, the temperature variations of the carbonized sorghum straws with different heights were monitored in real-time. The temperature of the evaporation interface of the carbonized sorghum straw (1 cm in height) increased from 23.7 °C to 29.2 °C within 1 h (Figure S6); however, an increase in the height of the carbonized sorghum straw led to a lower surface temperature. The stable surface temperature was only 26.2 °C for the 8 cm-tall carbonized sorghum straw. The lower surface temperature avoided dissipating heat to the environment and thus brought a tiny energy loss and higher energy conversion efficiency [47,48]. Therefore, the optimized height of the carbonized sorghum straw was 8 cm.

### 3.3. Photothermal and Solar Evaporation Properties of the Carbonized Sorghum Straw

The photothermal property of carbonized sorghum straw was evaluated by an IR camera. The surface temperatures of the wet carbonized sorghum straw increased from ~22.0 °C to ~26.2 °C, 28.8 °C, 30.6 °C, and 35.8 °C under 1 to 4 Sun illumination, respectively (Figure 4c and Figure S7). However, the temperatures of the dry carbonized material rapidly enhanced from ~30.0 °C to ~40.7 °C, 53.0 °C, 62.3 °C, and 80.3 °C under 1 to 4 Sun illumination, respectively (Figure 4d and Figure S8). The surface temperatures of the dry carbonized sorghum straw were higher than that of the wetted carbonized sorghum straw under the same illumination conditions at the same time interval. The lower temperature of the wetted carbonized sorghum was attributed to the rapid evaporation rate.

Encouraged by the results of great absorbance, prominent thermal barrier, superior hydrophilicity, and prominent photothermal conversion properties, the carbonized sorghum straw was evaluated as a solar-driven water purification device. To methodically investigate the performance of the evaporation device derived from carbonized sorghum straw, an 8 cm-height carbonized sorghum straw was investigated under 1 Sun illumination. The evaporation performance was obtained by monitoring the mass changes of water (Figure 4e). Due to the unique vascular bundle structure, parenchyma cells, and abundant pore structure, the carbonized and natural material evaporation rates were 3.173 kg m^−2^ h^−1^ and 1.819 kg m^−2^ h^−1^, respectively, which were 8.5 times and 4.9 times higher than that of pure water (0.372 kg m^−2^ h^−1^), respectively. The evaporation rates of carbonized materials, natural, and pure water without illumination were also investigated to exclude the environmental influence (Figure 4f). The evaporation rates of the pure water, natural, and carbonized sorghum straw were 0.256, 1.192, and 1.569 kg m^−2^ h^−1^, respectively.

**Figure 5:**
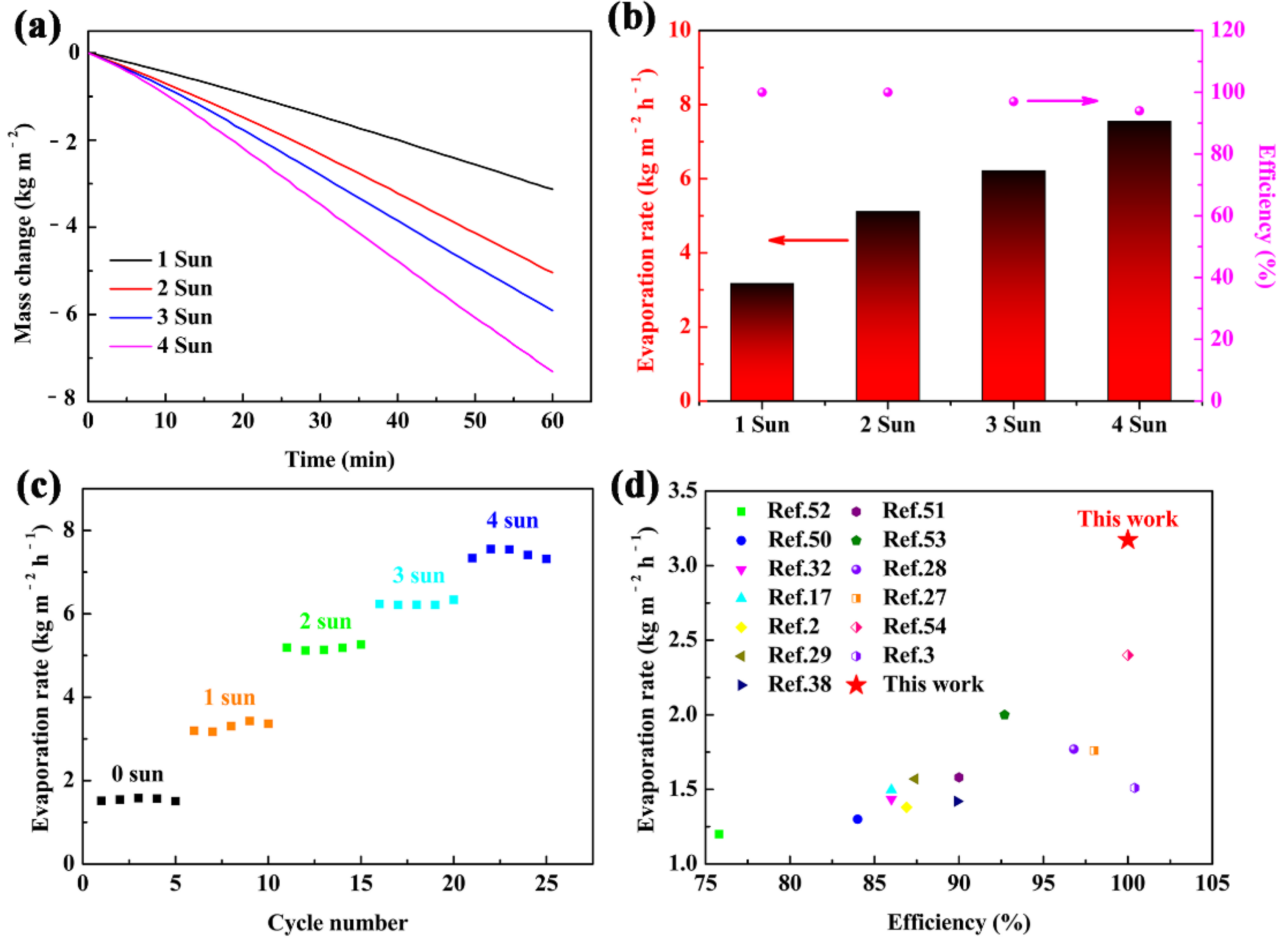
(**a**) Mass changes; (**b**) efficiencies and evaporation rates of carbonized sorghum straw under 1–4 Sun illumination; (**c**) cycling performances of the carbonized sorghum straw under 1–4 Sun illumination, each cycle kept for 1 h; (**d**) evaporation rate and efficiency of carbonized sorghum straw compared with previous reports under 1 Sun illumination.

The evaporation performance of the carbonized sorghum straw under higher optical intensity was further investigated. The evaporation rates were 5.119, 6.214, and 7.554 kg m^−2^ h^−1^ under 2, 3, and 4 Sun illumination, respectively (Figure 5a). The energy conversion efficiencies under different optical concentrations were 100%, 100%, 97%, and 94% for 1, 2, 3, and 4 Sun, respectively (Figure 5b). The variation tendency of the energy conversion efficiency was inconsistent with that of the evaporation rate. This result was considered to be due to the water evaporating rate being faster than the water drawing rate on the interface under the high optical intensity, leading to the surplus thermal diffusion to the side paths and environment [32,49]. The reusability of the carbonized sorghum straw was also evaluated under 1–4 Sun illumination, each cycle maintained 1 h (Figure 5c). The carbonized sorghum straw showed a steady evaporation rate, indicating its outstanding reusable ability. Compared with the previously reported solar evaporators, the carbonized sorghum-straw-based evaporator exhibited a superior performance in terms of the evaporation rate and energy conversion efficiency (Figure 5d) [2,3,17,27,28,29,32,38,50,51,52,53,54]. The structure and light absorption properties were important for the evaporating ability of the solar evaporator. The prominent energy conversion efficiency and evaporation rate benefitted from the unique vascular bundle structures, superior hydrophilicity, great absorbance, prominent thermal barrier endowed by parenchyma cells, and excellent photothermal conversion properties of the carbonized sorghum straw. The unique vascular bundle structures and superior hydrophilicity accelerated the water absorption and transfer process, while the high absorbance, good thermal insulation, and excellent photothermal conversion properties greatly reduced heat loss and focused the heat on the surface. Therefore, the carbonized sorghum-straw-based evaporator demonstrated the highest energy conversion efficiency and evaporation rate. The additional advantage of the carbonized sorghum straw is that it is low-cost and can be easily applied in practice.

### 3.4. Solar Desalination Performance and Potential Application of the Carbonized Sorghum Straw

**Figure 6:**
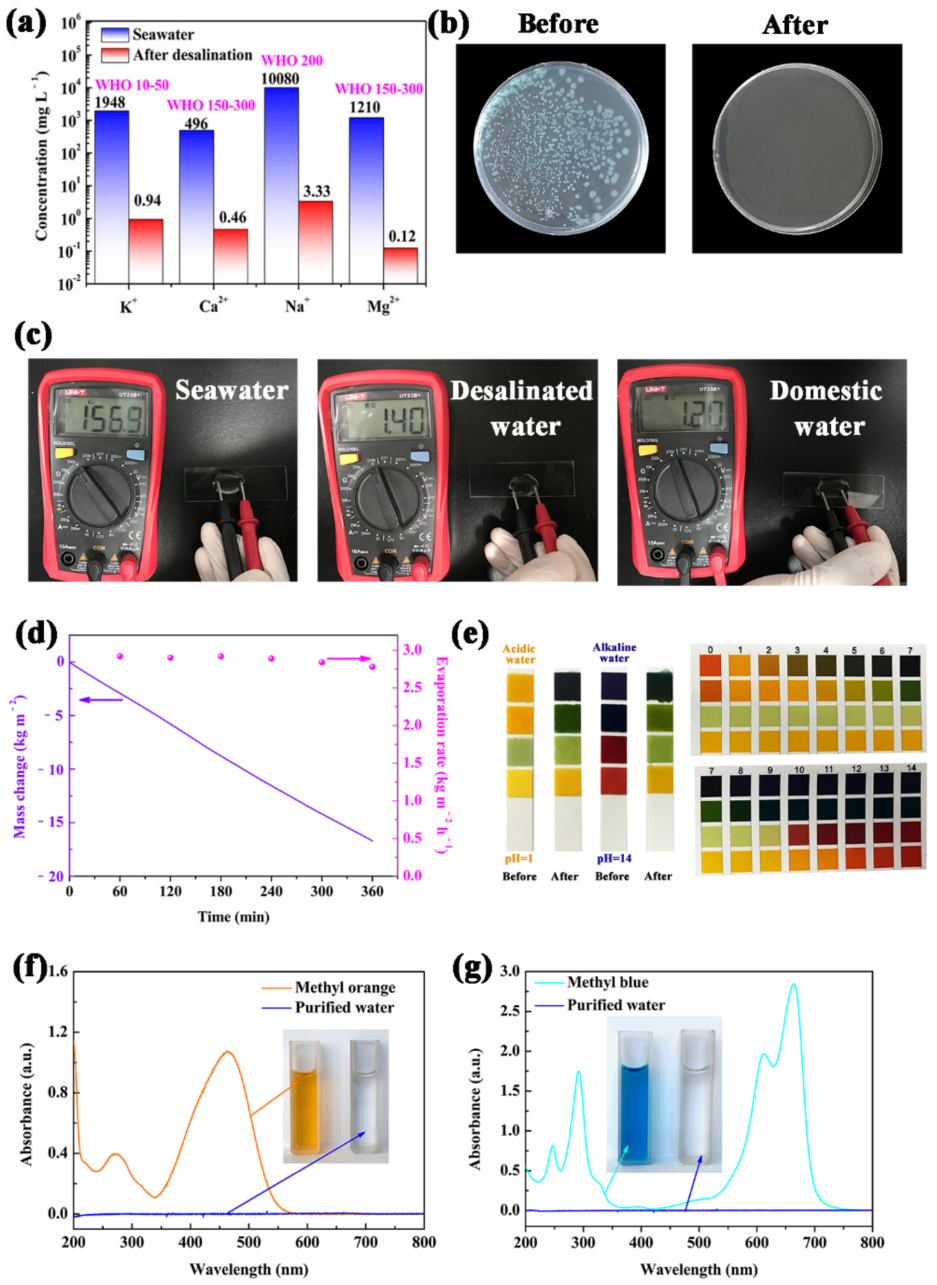
(**a**) ICP-MS results of BoHai seawater and desalinated water. (**b**) Images of tryptone agar plate incubated with real seawater sample before and after distilled. (**c**) Electrical resistances measurements of water samples by multimeter (the distance between electrodes remains the same). (**d**) Mass change and evaporation rate under 1 Sun illumination in 20 wt% NaCl. (**e**) Images of pH test strips added strong acid/alkaline aqueous before and after purification. The UV−vis spectra of (**f**) MO and (**g**) MB simulated wastewater before and after purification; inset photographs: color changes before (left) and after (right) solar purification.

To investigate the potential application of the carbonized sorghum straw in solar desalination, real seawater was collected from the BoHai sea. The ion concentrations of the distilled water produced by the carbonized sorghum straw were measured by inductively coupled plasma–mass spectrometry (ICP-MS). Figure 6a exhibits the concentration changes of Ca^2+^, K^+^, Mg^2+^, and Na^+^ before and after solar desalination. All ion concentrations were largely reduced after desalination (Ca^2+^: 0.46 mg L^−1^; K^+^: 0.94 mg L^−1^; Mg^2+^: 0.12 mg L^−1^; Na^+^: 3.33 mg L^−1^), which was much lower than the specified values of the World Health Organization (WHO) for drinking water, proving that the carbonized sorghum-straw-based evaporator has good desalination ability.

The safety of the distilled water was further evaluated by coating before and after distillation seawater sample onto the tryptone agar plates (Figure 6b). Many bacterial colonies are found on the tryptone agar plate cultured with the original seawater. However, no bacterial colony was observed in the treated seawater. These results demonstrate that the bacteria can be removed during the progress of desalination. In addition, the electrical resistances of the desalinated water can directly reflect the water quality [44]. The electrical resistances of seawater, desalinated water and domestic water (cooled, boiled water) were measured to intuitively evaluate the purity of the desalinated water (Figure 6c). The electrical resistances are 1.40 MΩ, 156.9 KΩ and 1.20 MΩ for desalinated water, seawater and domestic water, respectively. The highest electrical resistance suggests the effective purification of seawater and the obtained water can be used as freshwater.

To further explore the ability of resistance to salt crystallization during the purification process, salt tolerance tests were conducted with NaCl (20 wt%) for 6 h under 1 Sun illumination. Images were acquired at hourly intervals to record the situation of NaCl crystallized on the surface of the carbonized sorghum straw. After 6 h, no macroscopical NaCl crystals were observed on the heating surface of the carbonized sorghum straw during the whole process (Figure S9). The time-dependent mass change and evaporation rate of the carbonized sorghum straw in 20 wt% NaCl were investigated (Figure 6d). There was no obvious decrease during the whole evaporation process. Such results demonstrate that the carbonized sorghum straw has a high resistance to salt crystallization, which is conducive to long-term solar desalination.

To fully exploit the practical applications of the carbonized sorghum straw, strong acid (0.5 M H_2_SO_4_, pH = 1.0) and alkali aqueous (1 M NaOH, pH = 14.0) solutions were chosen as the water source. The pH values of purified water obtained from acid or alkali aqueous were all close to neutral (pH ≈ 7.0), indicating acid and alkali resistance of the carbonized sorghum straw (Figure 6e).

To evaluate the feasibility of the carbonized sorghum straw for wastewater-containing organic pollutants, methyl blue (MB) and methyl orange (MO) were chosen as the model contaminants and added in the deionized water for water purification experiments. Notably, the purified water became colorless and transparent via the present purification process; meanwhile, the characteristic peaks of MO and MB at 464 and 664 nm were invisible for the purified water (Figure 6f,g). These results suggest that the carbonized sorghum straw has excellent ability to purify water containing organic dyes. All results indicate that the carbonized sorghum straw has a broad application prospect in solar desalination and wastewater purification.

**Figure 7:**
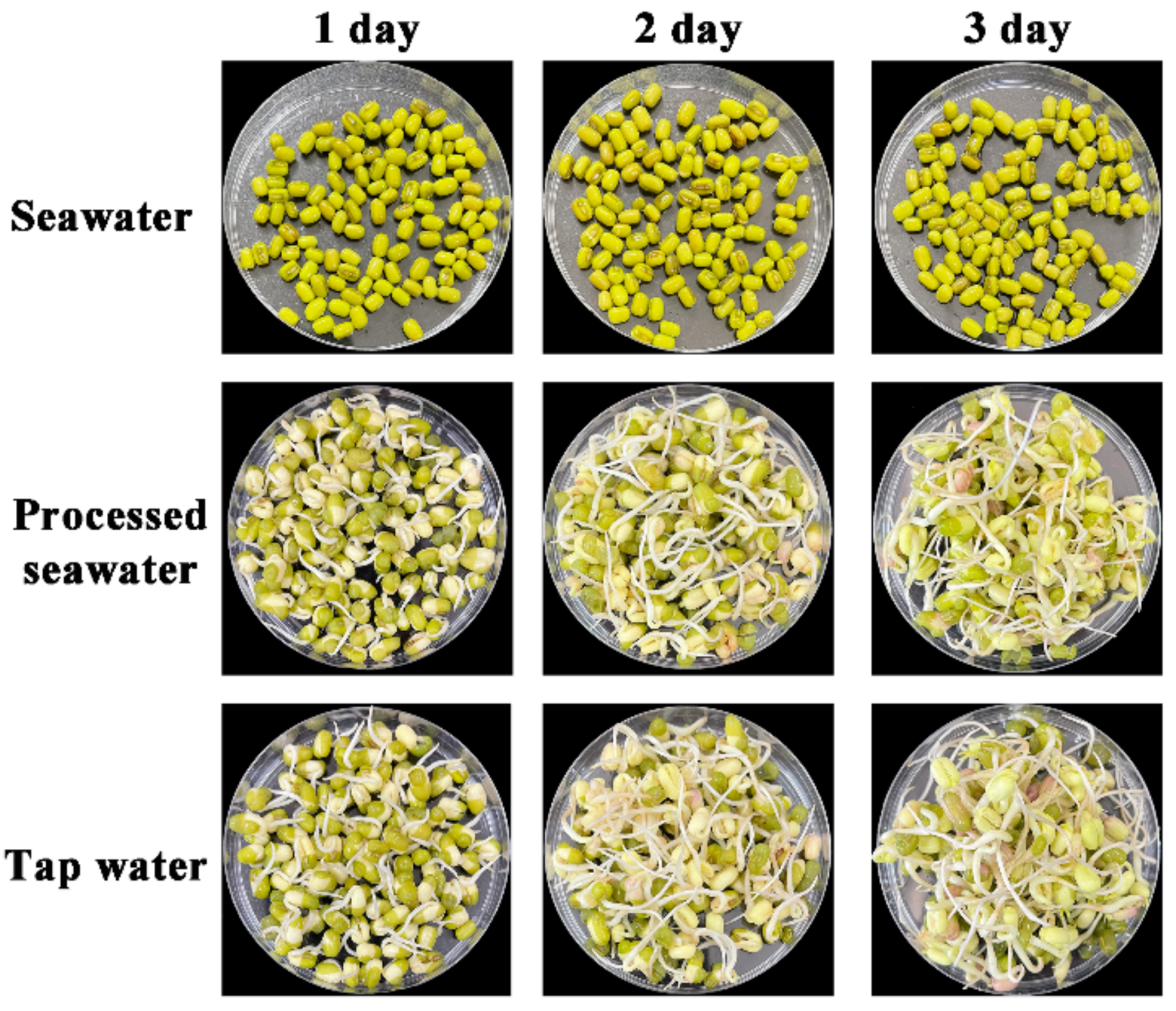
Images of mung bean sprouts cultured with different water sources.

To investigate the practical application in food production, the processed seawater was directly applied for producing mung bean sprouts. One hundred mung beans were soaked in BoHai seawater, tap water, and processed seawater. Mung beans became plump and burst the seed coat in the tap water and processed seawater after 24 h; however, mung beans soaked in BoHai seawater only puffed up slightly (Figure S10). After soaking, the growth of the mung beans covered with gauze moistened with different water sources was observed. The germination rates of the mung beans in BoHai seawater, processed seawater, and tap water were 0%, 99%, and 100%, respectively. The morphologies of mung bean sprouts had no noticeable difference in the case of tap water and processed seawater during the cultivation process (Figure 7).

## 4. Conclusions

A solar-driven evaporator-based on biochar was constructed and utilized to produce drinking water. The unique structure (multi-level pore bundle for water supply and the parenchyma cells for thermal barrier) and efficient solar absorption of biochar endow the present evaporator with the prominent performance. The energy conversion efficiency and evaporation rate are 100% and 3.173 kg m^−2^ h^−1^ under 1 Sun illumination, respectively, which surpass the previously reported biomass-derived solar evaporators (carbonized *Enteromorpha prolifera*, 1.3 kg m^−2^ h^−1^, 84%; carbonized lotus seedpods, 1.3 kg m^−2^ h^−1^, 86.5%; carbonized daikon, 1.57 kg m^−2^ h^−1^, 85.9%; carbonized corncob1.434 kg m^−2^ h^−1^, 86%; carbonized sunflower heads, 1.51 kg m^−2^ h^−1^, 100.4%) [3,28,36,37,38]. The constructed evaporator demonstrates potential application in the fields of seawater desalination, acidic/basic water purification, and organic polluted water treatment. The processed water can be directly used to food production. All these results suggest that the constructed evaporator based on abundant raw materials has potential in the field of sustainable food.

## Data Availability

Not applicable.

## Supplementary Materials

The following are available online at https://www.mdpi.com/article/10.3390/foods10123087/s1, Figure S1: Optical microscope images of natural sorghum straw. (a–c) Top view and (d–f) cross view of the sample. Figure S2: Pore size distribution of the carbonized sorghum straw measured with mercury-injection method. Figure S3: Reflectance spectra of the natural and carbonized sorghum straw. Figure S4: Contact angles of the carbonized sorghum straw. Figure S5: Water pumping with a carbonized sorghum straw (2 cm) and distribution through on the filter paper (5 cm in diameter). Figure S6: Infrared thermal images of carbonized sorghum straw under 1 Sun illumination. Figure S7: Infrared thermal images of wet carbonized sorghum straw under different illumination. Figure S8: Infrared thermal images of dry carbonized sorghum straw under different illumination. Figure S9: Photographs of carbonized sorghum straw testing in 20 wt% NaCl under 1 kW m^−2^ for 6 h. Figure S10: Images of mung beans before and after soaking in different water sources.
