# Supplementary material for: Efficient Solar-Driven Water Purification Based on Biochar with Multi-Level Pore Bundle Structure for Preparation of Drinking Water
Source: Foods. 2021 Dec 13;10(12):3087. doi: 10.3390/foods10123087 (PMC8701808; doi:10.3390/foods10123087)
Supplement: Supplementary file 1 [file foods-10-03087-s001.zip › foods-1447752-SI.pdf]

Supporting Information

Efficient Solar-Driven Water Purification Based on Biochar with Multi-level Pore Bundle Structure for Preparation of Drinking Water

Zhen Zhang[1], Shizheng Jiang [1], Hannan Chen [1], Hao Qi [1], Yali Chen [1], Yujie Chen [1], Qiliang Deng [1,*] and Shuo Wang[1,2,*]

[1.] State Key Laboratory of Food Nutrition and Safety, Key Laboratory of Food Nutrition and Safety, Ministry of Education of China, Tianjin University of Science and Technology, Tianjin 300457, China.

[2.] Tianjin Key Laboratory of Food Science and Health, School of Medicine, Nankai University, Tianjin 300071, China.

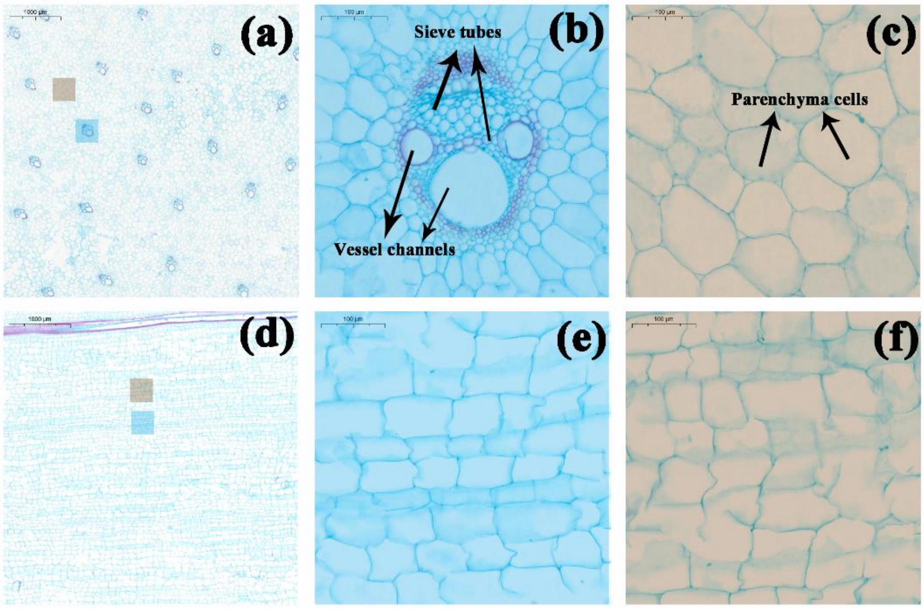

Figure S1. Optical microscope images of natural sorghum straw. (a-c) Top view and (d-f) cross view of the sample. Scale bars: 1000 μm (a, d); 100 μm (b, c, e, and f).

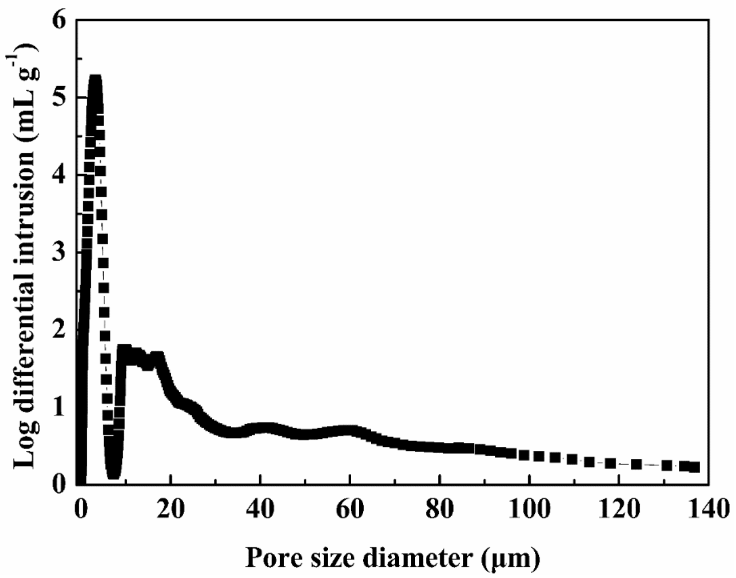

Figure S2. Pore size distribution of the carbonized sorghum straw measured with mercury-injection method.

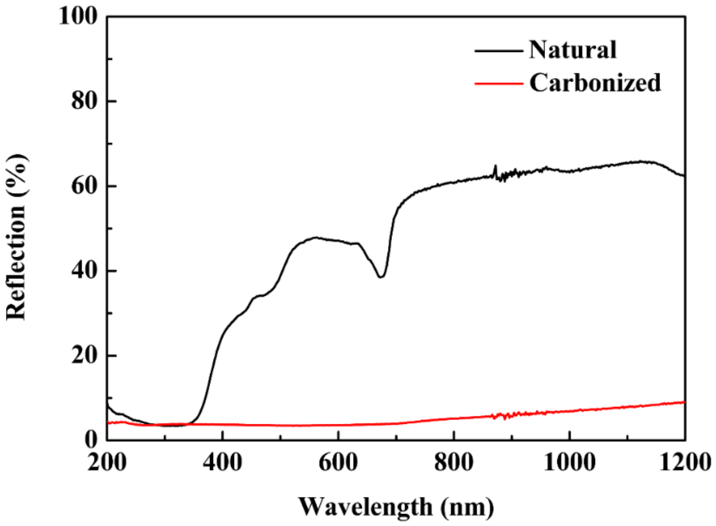

Figure S3. Reflectance spectra of the natural and carbonized sorghum straw.

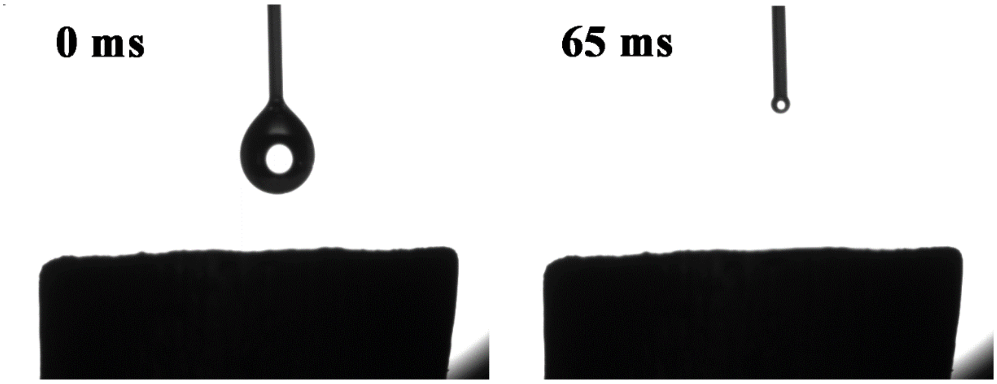

Figure S4. Contact angles of the carbonized sorghum straw.

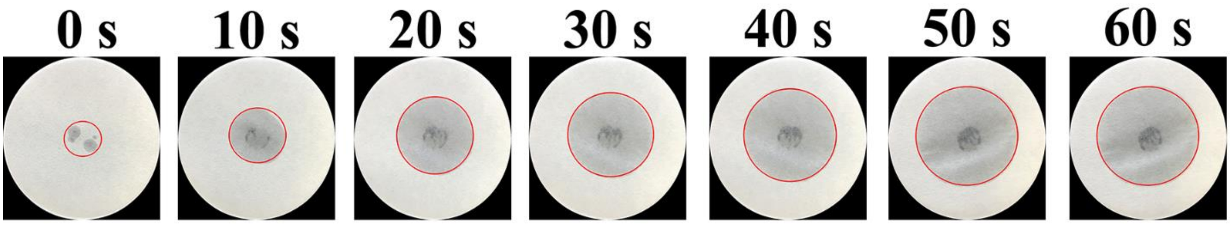

Figure S5. Water pumping with a carbonized sorghum straw (2 cm) and distribution through on the filter paper (5 cm in diameter).

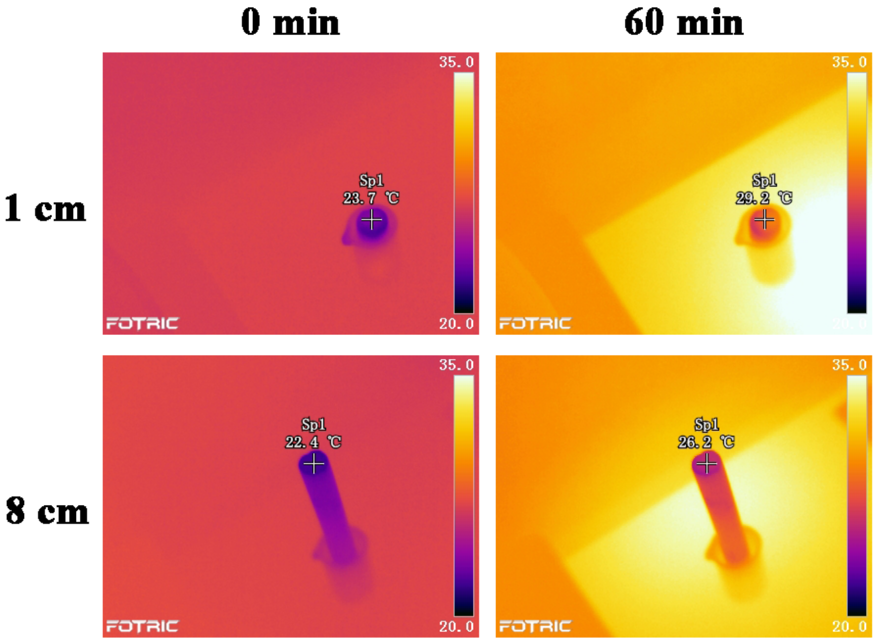

Figure S6. Infrared thermal images of carbonized sorghum straw under 1 Sun illumination.

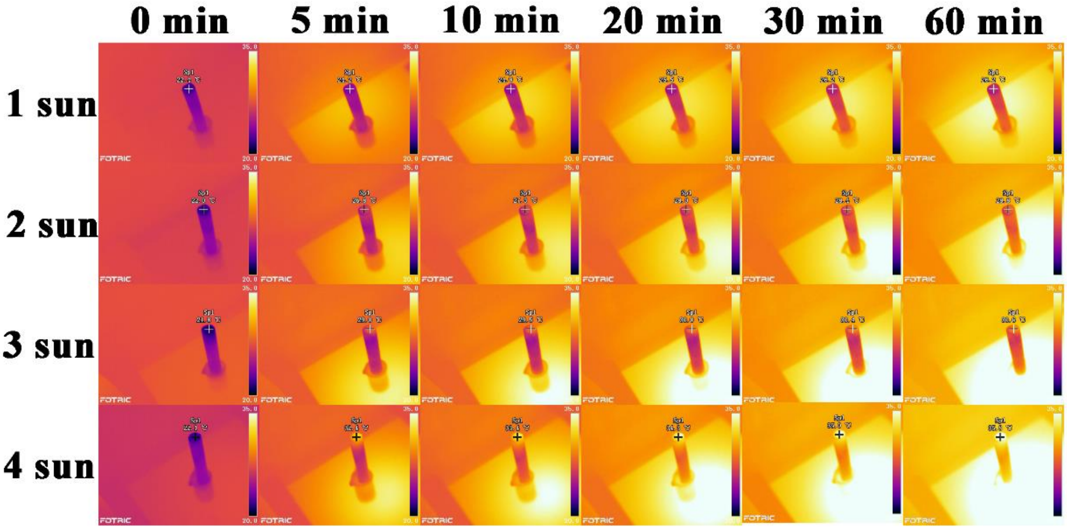

Figure S7. Infrared thermal images of wet carbonized sorghum straw under different illumination.

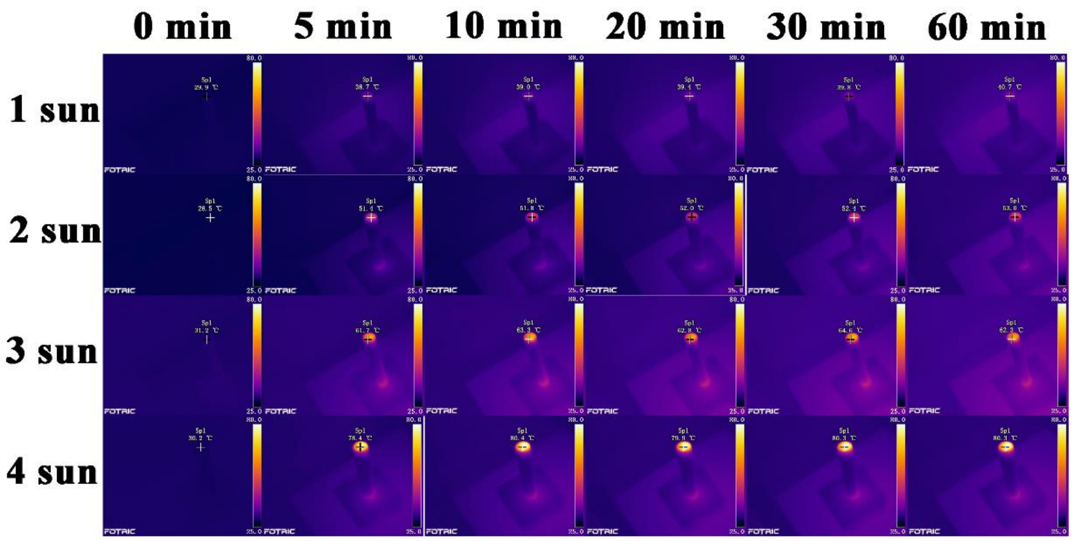

Figure S8. Infrared thermal images of dry carbonized sorghum straw under different illumination.

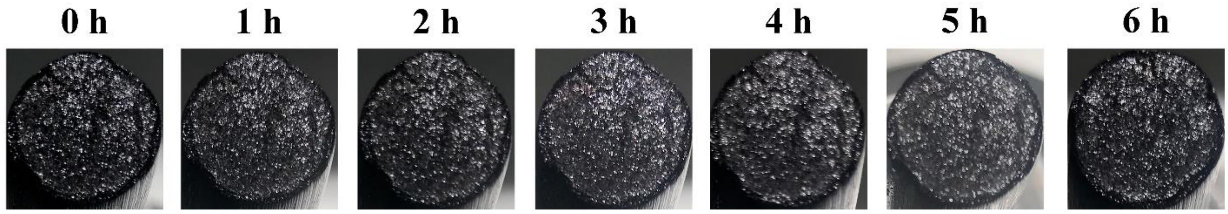

Figure S9. Photographs of carbonized sorghum straw testing in 20 wt% NaCl under 1 kW m^{-2} for 6 h.

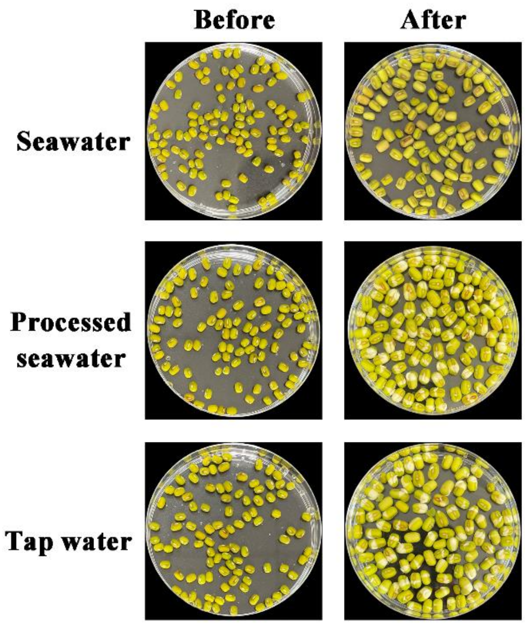

Figure S10. Images of mung beans before and after soaking in different water sources.
